# Association between Loneliness and Memory Function through White Matter Hyperintensities in Older Adults: The Moderating Role of Gender

**DOI:** 10.3390/bs13100869

**Published:** 2023-10-23

**Authors:** Hyeyoung Park, Hairin Kim, Seyul Kwak, Yoosik Youm, Jeanyung Chey

**Affiliations:** 1 Department of Psychology, Seoul National University, Seoul 08826, Republic of Korea; phyeyoung1114@gmail.com (H.P.); hairin.kim@gmail.com (H.K.); 2 Department of Psychology, Pusan National University, Busan 46241, Republic of Korea; sykwak@pusan.ac.kr; 3 Department of Sociology, Yonsei University, Seoul 03722, Republic of Korea; yoosik@yonsei.ac.kr

**Keywords:** loneliness, white matter lesion, cognitive function, clinical neuropsychology, gender difference

## Abstract

Loneliness has an important impact on memory function in late life. However, the neural mechanism by which loneliness detrimentally influences memory function remains elusive. Furthermore, it remains unclear whether the association between loneliness and memory function varies by gender. The current study aimed to investigate the neural mechanism underlying the association between loneliness and episodic memory function and explore whether it varies with gender among cognitively normal older adults. A total of 173 community-dwelling adults aged 60 years or older from the Korean Social Life, Health, and Aging Project (KSHAP) study (mean age = 71.87) underwent an assessment of loneliness, neuropsychological testing, and structural magnetic resonance imaging. The association between loneliness and episodic memory function was mediated by the volume of white matter hyperintensities (WMHs), but not by hippocampal or gray matter volumes. In addition, the association between loneliness and memory function through WMHs was significantly moderated by gender; specifically, the indirect effect was significant among men but not among women. The study suggests that WMHs may be a potential neurological mechanism that causes late-life memory dysfunction associated with loneliness in older men. The findings underscore the need for gender-specific interventions to mitigate memory impairment associated with late-life loneliness, with significant public health implications.

## 1. Introduction

Loneliness is defined as a distressing feeling associated with discrepancies between an individual’s actual and desired social relationships [1]. Accumulating evidence has suggested that loneliness has a detrimental effect on cognitive health in late life, specifically on various domains of cognitive function, including episodic memory and executive function [1,2,3,4]. A longitudinal study found that higher degrees of loneliness are associated with double the risk of developing Alzheimer’s disease (AD) at follow up [5].

Although a consistent body of literature has identified the association between loneliness and cognitive function, several important questions remain unanswered. First, the neural mechanism by which loneliness detrimentally influences cognition is elusive. Understanding the neural mechanism is crucial, as it may clarify the potential intervention that could prevent or delay the onset of loneliness-induced cognitive impairment. Therefore, we were interested in investigating whether brain pathological and anatomical markers of cognitive decline play a specific role in the loneliness-related cognitive deterioration.

A longitudinal study found that increases in loneliness were correlated with a greater volume of white matter hyperintensities among non-demented older adults [6,7,8]. White matter hyperintensities, a marker of small-vessel cerebrovascular disease observed on T2-weighted magnetic resonance imaging in older adults, are a valid predictor of episodic memory. Moreover, it has been known that WMHs confer additive risk for AD and predict the progression of AD-related neurodegeneration [9,10,11]. Given these findings, we chose white matter hyperintensities as a candidate mechanism underlying loneliness-related memory function. Moreover, lonely older adults had lower gray matter and hippocampus volumes, which are among the strongest radiological predictors of memory deficits, compared with those who were not lonely [7,12]. Accordingly, we also examined whether whole gray matter and hippocampus volume play a specialized role in the relationship between loneliness and memory performance as a mediator.

One important factor to consider when examining the link between loneliness and cognition is gender. Numerous studies highlighted gender differences in physiological responses to social stress [13,14]. Specifically, men with low social integration were at a higher risk of mortality than women and the feeling of loneliness was associated with elevated inflammation only in men [13,15,16,17]. Considering that lonely men are more vulnerable to disease and death than lonely women, the same level of loneliness might also be expected to induce more adverse cognitive outcomes in men than women. However, few studies to date have focused on gender differences in the association between loneliness and cognitive functions. Therefore, the current study explored whether loneliness-related memory functions through neurological factors differ by gender.

Lastly, it is crucial to distinguish between subjective and objective features of social isolation. Because loneliness is a subjective experience related to unfulfilled social needs, it is conceptually distinct from objective social isolation, such as a small social network size and infrequent social interaction. Despite the distinction, most existing studies have used the inconsistent terminology of indicators of social isolation and loneliness [18,19,20]. As such, inconsistent conceptual terminology can interrupt communication between researchers and limit knowledge about the comparative effects of different social relationship dimensions. Therefore, we separately assessed two distinct aspects of social isolation, and the influences of loneliness were examined after controlling the effects of objective features of social isolation in all analyses.

**Figure 1:**
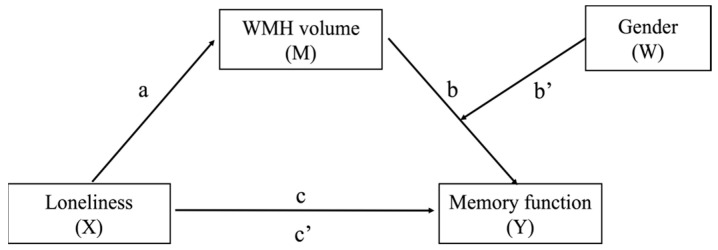
A hypothetical model. Path a = the effect of X on M; Path b = the effect of M on Y; Path c = the total effect of X on Y; Path b’ = interaction between M and W; Path c’ = direct effect of loneliness on memory function; Path (a) (b + b’W) = conditional indirect effect of X on Y through M.

Therefore, this study aimed to identify neural mechanisms underlying the association between loneliness and memory functioning and examine the moderating role of gender in this relationship. We hypothesized that white matter hyperintensities and hippocampal and whole gray matter volumes would mediate the relationship between loneliness and memory performance as a candidate mechanism. We also hypothesized that the indirect mediating effects of loneliness on memory function through neurological factors would be stronger in men than women. Figure 1 presents a hypothetical model.

## 2. Materials and Methods

### 2.1. Participants

An a priori power analysis was performed using GPower 3.1 to determine the required number of participants to test linear regression analyses. The parameters used were as follows: 0.80 power, alpha 0.05, six predictors, and a medium effect size (*f^2^* = 0.15) of the hypothesized linear regression analyses based on similar studies [2]. The results indicated a required sample size of at least 98 participants. To allow for anticipated dropout (20%), a minimum sample size of 118 was targeted. The study used data from the Korean Social Life, Health, and Aging Project (KSHAP), which was conducted in Township K and L located on Ganghwa Island, South Korea [21]. Initially, 418 older adults underwent neuropsychological tests and psychosocial surveys. Among these participants, those with the presence of psychiatric or neurological disorders, vision or hearing problems, having a history of losing consciousness due to head trauma, hypertension or diabetes uncontrollable with drugs or insulin, and metal in the body that cannot be removed were excluded based on the Health Screening Exclusion Criteria [22]. In addition, older adults who were highly suspected of dementia were excluded based on age and education-stratified norm (<−1.5 SD) of the Mini-Mental State Examination for Dementia Screening (MMSE-DS) [23] and the Clinical Dementia Rating (CDR). Based on the dementia rating, participants with mild cognitive impairment (CDR ≥ 0.5) were excluded. Among participants who did not meet the exclusion criteria (n = 359), 206 older adults were available for MRI acquisition. Participants were instructed to abstain from alcohol consumption the day before the experiment and from smoking and caffeine in the morning of the MRI scan to control for the effects of substances. All the participants completed the MRI scan in the morning. Among the participants who completed the entire imaging protocol, those who had either diffuse old infarcts or excessive head motion during the scans were excluded. In total, 173 healthy older adults aged 60 to 93 years (n = 102, women; M_age_ = 71.87 and SD_age_ = 6.79) were included in the final dataset. The study design and procedure were approved by the Institutional Review Board of Seoul National University (IRB No. 1801/001-003) and Yonsei University (IRB No. 1040917-201501-HRBR-100-04). All participants provided a written informed consent for the research procedure.

### 2.2. Loneliness

Loneliness was assessed with the Korean version of the revised UCLA loneliness scale (UCLA-r) [24,25]. The revised UCLA Loneliness Scale consists of 20 items that assess perceptions of loneliness. Participants were asked to rate each item from 1 (Never) to 4 (Often) to assess how often they agreed with the description. Total scores on the UCLA-r ranged from 20 to 80 and higher scores reflect greater loneliness. Cronbach’s alpha across all 20 items ranged from 0.86 to 0.87.

### 2.3. Social Network Measures

Objective social isolation can be characterized by lack of contact with others, as the opposite of integration. To assess objective social isolation, a questionnaire comprising three items was administered: living alone, social network size, and average frequency of interaction with network members [18,26]. First, the individual’s social network size was established using a name generator. During face-to-face interviews conducted by trained interviewers, each participant was instructed to provide the names or nicknames of up to five close individuals to whom they most commonly discuss important things. Further, respondents were instructed to provide the following information: “how often do you meet directly with your discussion member?”. The questionnaire was rated on a scale of 1 (less than once a year), 2 (once a year), 3 (several times a year), 4 (once a month), 5 (once every two weeks), 6 (once a week), 7 (several times a week), and 8 (every day). The average score of an individual’s response was assessed to obtain the mean frequency of interaction with their network members. Marital status was binary coded according to whether the respondent was living with or without a spouse. Each provided response was included as a covariate in all analyses.

### 2.4. MRI Acquisition and Preprocessing

We obtained T1-weighted magnetic-prepared rapid-gradient echo (MPRAGE) and T2-fluid-attenuated inversion recovery (FLAIR) images using the 3.0-Tesla MAGNETOM Trio 32 channel coil at Seoul National University Brain Imaging Center. The T1-weighted images were acquired with the following scanning parameters: TR = 2300 ms, TE = 2.3 ms, FOV = 256 × 256 mm, and FA = 9°. T2-weighted FLAIR images were acquired with the following parameters: TR = 9000 ms, TE = 93.0 ms, FA = 150°, FOV = 220 mm, voxel size = 0.9 × 0.9 × 3.5 mm, and gap = 1.5 mm.

MRI findings were pre-processed using tools implemented in the Statistical Parametric Mapping software (SPM12; Wellcome Department of Imaging Neuroscience, Institute of Neurology, London, the UK) and were executed in MATLAB version r2020b (The MathWorks Inc.). White matter hyperintensities (WMHs) were segmented using T1-weighted images and T2-FLAIR images using a lesion prediction algorithm with the LST toolbox version 2.0.13 for SPM12 (www.statistical-modelling.de/lst.html). The estimated volume of WMHs was logarithmically transformed to adjust positive skewness in the distribution. T1-weighted images were bias-corrected and segmented using SPM12. The whole GM volumes and bilateral hippocampal volumes were proportionally adjusted with the total intracranial volume (ICV).

### 2.5. Neuropsychological Assessment

The Elderly Memory Disorder Scale (EMS) [27] was administered to assess the episodic memory of participants. EMS, a standardized neuropsychological battery, is a tool for assessing both verbal and nonverbal memory function in Korean older adults with less exposure to formal education. The EMS includes the Elderly Verbal Learning Test (EVLT), the Story Recall Test (SRT), and the Simplified Rey Figure Test (SRFT). The EVLT is a word-list learning task based on the California Verbal Learning Test [28], that measures verbal learning and memory. Participants were presented with a list of nine words from three categories over five trials. In each trial, the participant was asked to learn and immediately recall the items. The immediate recall test was followed by the long-term delayed recall test and the recognition test, in order. The SRT is a task based on the Logical Memory task of the Wechsler Memory Scales version III [29], in which the participant is told a short story about a kidnap that contains 24 semantic units and 6 theme units. Participants were immediately asked to memorize and recall the story in as much detail as possible. After a delay of 20 to 30 min, the delayed recall test was administered and the recognition test was administered lastly. The SRFT from the Geriatric Evaluation of Mental Status [30], which was used to assess visuospatial construction and spatial memory function, is a simplified version of the Rey–Osterrieth Complex Figure Test for older adults. Participants were asked to copy a figure composed of simple geometric features on a blank piece of paper and then immediately draw the figure from memory without seeing it. After a delay of 20 to 30 min, the delayed recall and the recognition tests were conducted. The long-term memory (LTM) recall index and the LTM recognition index were calculated from the performance on the three-memory test. The LTM Recall Index was calculated by dividing each delayed recall score on the EVLT, the SRT, and the SRFT by its maximum score and then summing them all. The LTM Recognition Index was calculated in an identical method using the correct delayed recognition scores on the three tests.

### 2.6. Statistical Analysis

Version 26.0 of SPSS (IBM corporation, Armonk, NY, USA) and PROCESS macro 3.5 [31] were utilized to test the research hypotheses. First, a descriptive statistical analysis of men and women was conducted using the independent *t*-test for continuous variables and Fisher’s exact test for categorical variables. In addition, Pearson correlation analysis was performed not only to describe the associations among the variables of interest and demographics, but also to determine whether they should be registered as covariates in the following analysis. Subsequently, model 4 of PROCESS macro for SPSS was used to examine whether volume of white matter hyperintensities (WMHs) mediates the relationship between loneliness and memory function (LTM Recall Index, LTM Recognition Index). Additionally, considering that previous studies reported whole gray matter (GM) and hippocampal volumes as possible predictors of late-life memory impairment [32,33], we also evaluated each of these variables as mediators in mediational analyses.

To test the second hypothesis, we examined the moderating role of gender in the indirect effects of loneliness-related memory function through neural mechanism. However, there was no prior hypothesis on which specific pathway would be affected by gender, as no other similar studies have been conducted so far. Therefore, as an exploratory approach, we initially examined gender differences in each pathway of the indirect effects (X→M or M→Y) through moderation analysis. We then included gender as the proposed moderator variable in the mediation models and tested the moderated mediation hypothesis with model 14 of PROCESS macro for SPSS. Bootstrap confidence intervals (CI) using 5000 random resamples were conducted to determine whether the mediating effect from Model 4 and moderating effect from Model 14 were significant. Age, educational level, and measures of objective social isolation were included as covariates for all analyses.

## 3. Results

### 3.1. Descriptive Statistics

Descriptive statistics for demographic, social network, neuroimaging, and cognitive variables among men (n = 71) and women (n = 102) are presented in Table 1. The two subsamples were similar in age, but women had fewer years of education than men. Men were more likely than women to live with their spouses. The two groups did not differ significantly in loneliness, social network size, and frequency of interaction with network members. For total volume of white matter hyperintensities (WMHs), after adjusting for age, years of education, and total intracranial volume, men had significantly greater WMHs volumes than women. No outliers in WMHs volumes were found in either men or women. Overall memory performance did not differ between men and women, after adjusting for age and education.

### 3.2. Correlation Analysis

Table 2 shows the results of the correlation analysis of the main study variables and demographic variables, including age and years of education. Gender showed significant correlation with years of education (r = 0.389, *p* < 0.001) and WMHs (r = 0.191, *p* = 0.01). Age showed positive correlation with WMHs (r = 0.531, *p* < 0.001) and negative correlation with both LTM indices (recall index: r = −0.371, *p* < 0.001; recognition index: r = −0.375, *p* < 0.001). Years of education showed positive correlation with both LTM indices (recall index: r = 0.349, *p* < 0.001; recognition index: r = 0.503, *p* < 0.001). Loneliness was significantly positively associated with WMHs (r = 0.154, *p* = 0.04) and negatively associated with both long-term memory (LTM) indices (recall index: r = −0.148, *p* = 0.048; recognition index: r = −0.17, *p* = 0.023). An individual’s depressive symptom was highly correlated with loneliness (r = 0.297, *p* < 0.001). WMHs were significantly negatively associated with both LTM indices (recall index: r = −0.404, *p* < 0.001; recognition index: r = −0.421, *p* < 0.001). All variables were checked for normality using the Kolmogorov–Smirnov test (loneliness: *p* = 0.735; log-transformed WMHs: *p* = 0.877; LTM recall index: *p* = 0.513; LTM recognition index: *p* = 0.491).

### 3.3. Simple Mediation Analysis

A series of mediation models were tested for both LTM Recall and Recognition indices, and the results of analyses are presented in Table 3. Each mediation analysis of memory function showed similar patterns. In both models, loneliness had a positive effect on WMHs volume (*B* = 0.009, *t* = 2.20, *p* = 0.029) and WMHs volume had a negative effect on LTM memory functions (Recall: *B* =−0.183, *t* = −2.844, *p* = 0.005; Recognition: *B* = −0.115, *t* = −3.458, *p* < 0.001). The direct effects of loneliness on memory functions were not significant (Recall: *B* = −0.005, *t* = −1.446, *p* = 0.150; Recognition: *B* = −0.003, *t* = −1.748, *p* = 0.082), but the mediating effect of WMHs volume on the relationship between loneliness and memory functions was statistically significant (Recall: *B* = −0.002, *SE* = 0.001, 95% CI = [−0.0041, −0.0001]; Recognition: *B* = −0.001, *SE* = 0.001, 95% CI = [−0.0022, −0.0001]). However, whole gray matter volumes did not significantly mediate the link between loneliness and memory functions (LTM Recall index: *B* = −0.0007, *SE* = 0.0008, 95% CI = [−0.0026, 0.0004]; LTM Recognition index: *B* = −0.0005, *SE* = 0.0004, 95% CI = [−0.0013, 0.0001]). Hippocampal volumes were also not a significant mediator of loneliness-related memory functions (LTM Recall index: *B* = 0.0005, *SE* = 0.0008, 95% CI = [−0.0008, 0.0024]; LTM Recognition index: *B* = 0.0002, *SE* = 0.0003, 95% CI = [−0.0003, 0.001]).

### 3.4. Moderation Analysis

**Figure 2:**
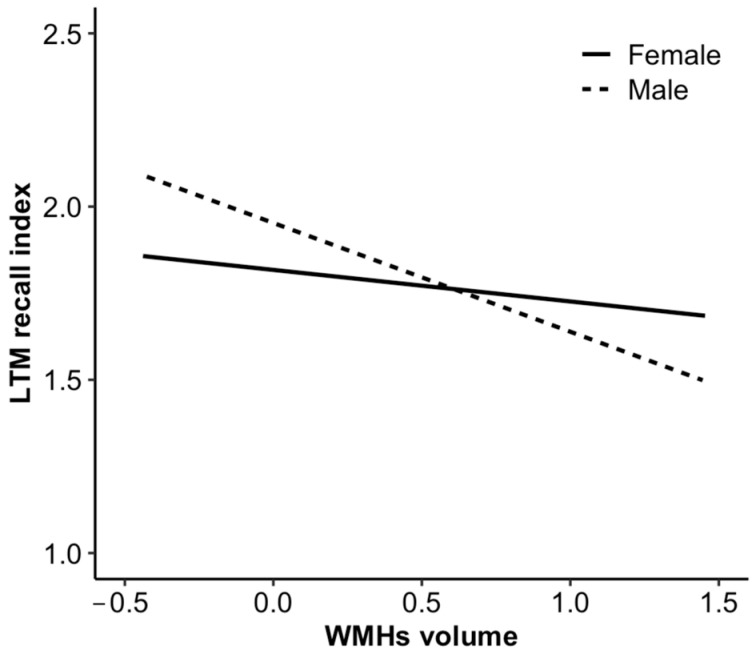
Simple slopes indicating the moderation effect of gender in the association between WMHs volume and LTM recall index.

Table 4 shows the results of the moderation analysis. The effects of loneliness on a volume of WMHs (B = 0.007, t =.871, *p* = 0.385) and the effects of WMHs volume on LTM recognition index (B = −0.071, t = −1.189, *p* = 0.236) were not significantly moderated by gender. However, the relationship between WMHs volume and LTM recall index was significantly moderated by gender (B = −0.270, t = −2.373, *p* = 0.018). In other words, the effect of WMHs volume on LTM recall memory function differed between men and women (Figure 2).

### 3.5. Moderated Mediation Analysis

Table 5 shows the results of the analysis that was conducted to identify the moderating effect of gender on the association between loneliness and LTM recall index through WMHs volume. Loneliness, volume of WMHs, and gender were not significant predictors of LTM recall index. However, the interaction of gender and volume of WMHs was a significant predictor of LTM recall (*B* = −0.259, *t* = −2.27, *p* = 0.03), indicating that gender moderated the mediation in the effect of loneliness on the LTM recall memory function through volume of WMHs. This finding was verified through bias-corrected nonparametric percentile bootstrapping. In the bootstrap test, the conditional indirect effect of loneliness on recall memory function through WMHs was significant for men (*B* = −0.003, *SE* = 0.002, 95% CI = [−0.007, −0.0001]), but not for women (*B* = −0.001, *SE* = 0.001, 95% CI = [−0.002, 0.001]) (Table 5 and Table 6).

## 4. Discussion

The current study investigated the neural mechanisms underlying loneliness-related memory deficits and examined whether these mediating effects vary by gender. Our findings showed that loneliness negatively affected memory performance through white matter hyperintensities (WMHs). Moreover, the study identified gender differences in these mediation effects, specifically the mediating effect of WMHs on the association between loneliness and recall memory function was significant in men, but not in women. These findings indicate that loneliness is more strongly associated with memory function through WMHs among men than among women.

In the mediation analysis, we found that increases in loneliness were associated with increased volume of WMHs, and those who have larger WMHs performed worse on the long-term memory recall and recognition tests. These results are consistent with a previous study that showed loneliness to be associated with the progression of WMHs [6,8]. Since lonely individuals have an increased sensitivity to social threats, they are more likely to experience negative thoughts and daily events as stressful [2,34]. Chronic experience of social stress may activate biological responses, such as the dysregulation of HPA functioning, hypercortisolism, and impaired immune functioning [12,35], and WMHs are one of the negative outcomes of prolonged HPA axis activation and abnormally elevated inflammatory response [36]. In addition, an aging study with neuroimaging reported that negative health outcomes of loneliness, such as cardiovascular risk factors and established cardiovascular disease, were strongly associated with the presence and progression of WMHs [37]. These previous studies support our findings that the volume of WMHs is a neural mechanism linking the association between loneliness and memory function.

The study found that gender moderates the mediating effect of WMHs on the association between loneliness and LTM recall index. The results showed that higher levels of loneliness were associated with a greater volume of WMHs and more severe memory deficits in men, but not in women. These findings extend existing knowledge on gender differences in the detrimental effects of loneliness on health in late life. While previous studies have only found gender differences in the relationship between loneliness and physical health, this study is the first to show that gender differences may also be present in the relationship between loneliness and late-life cognitive function. The moderated mediation results in the current study may be due to different patterns of social relationships across the gender. Previous research has shown that low levels of education and living alone are associated with a higher risk of social isolation and poor health in later life [38,39]. As shown in Table 1, women in this study are more likely to live alone and have lower levels of education than men. Although these statistics suggest that women are more likely to be socially isolated and to have poorer physical health than men, men had higher levels of WMH than women, and there was no significant difference in memory test scores between the genders. In other words, despite the differences in education and living alone, there were no significant differences in neuropathological burden and cognitive function across genders. These findings can be explained by stress buffering theory, which suggests that social support from intimate relationships can buffer the harmful effects of social challenges on health and promote psychological and physical well-being [40]. Evidence suggests that women tend to maintain more intimate partners and larger support networks, while men typically rely on their spouses for support in late life [41,42,43,44,45]. Abundant social support is known to be a protective factor, buffering the effects of neuropathological burden and delaying the onset of cognitive decline [43,46,47]. Therefore, older women may be less likely to experience loneliness-related white matter lesions leading to memory deficits compared to older men. The findings have important implications for the development of gender-specific interventions to mitigate the negative effects of loneliness on cognitive health.

The study found that gender differences were observed in the association between WMHs and memory function but not in the association between WMHs and loneliness. These findings suggest that other external variables related to WMHs, in addition to loneliness and WMHs, might have influenced the results. For example, men have a higher risk of cerebrovascular disease and hypertension than women, which are strongly associated with the development of WMHs [48,49,50]. In addition, men have higher rates of smoking and alcohol consumption compared to women, both of which are strongly associated with WMHs and cognitive decline [51,52]. These additional external variables may have interacted synergistically with WMHs to affect memory function, potentially explaining the observed gender differences in mediating effects.

The current study had several limitations. First, since it was conceptualized via a cross-sectional design, the causality cannot be inferred. For example, our findings do not rule out the possibility that loneliness may appear as the behavioral manifestation of cognitive dysfunction [1]. To minimize this possibility, the study excluded older adults with mild or significant cognitive impairment through screening procedures. According to other longitudinal study findings, loneliness could be a predictor of greater cognitive decline, but low cognition at baseline did not predict changes in loneliness over time [53]. A future replication study using longitudinal design is required to validate the causality between loneliness and memory deficits. Second, considering that participants in the study are older adults in a rural township, the findings could not be generalized to the overall population. Thus, the findings in this study should be interpreted carefully, and further research is needed for Korean older adults in urban area to obtain robust results.

In summary, the study found that white matter hyperintensities, a key risk factor for cognitive decline, to be a mechanism underlying the association between loneliness and memory function in older men. In other words, men who feel lonely are more likely to have a greater volume of white matter lesions and also to experience progression of long-term recall memory dysfunction compared to women in late life. These findings may have significant implications for public health, as understanding how loneliness affects memory performance may lead to the development of therapeutic and preventive interventions to address the loneliness-related memory deficits. Furthermore, gender differences in mediating effects can be used to develop gender-specific interventions for lonely older adults in the community.

## Figures and Tables

**Table 1:** Descriptive characteristics of the participants (N = 173).

Variables	Overall Sample		
	Men (n = 71)	Women (n = 102)	*p*
Age (y), M ± SD	72.65 (6.29)	71.32 (7.09)	0.09
Educational level (y), M ± SD	9.13 (4.19)	5.9 (3.77)	<0.01
Marital status, n (%)			
Not living alone	69 (97.18)	77 (75.49)	<0.01
Loneliness, M ± SD	37.32 (9.62)	35.39 (8.57)	
Social engagement, M ± SD			
Social network size	3.49 (1.62)	3.42 (1.57)	0.39
Interaction frequency	181.9 (99.67)	179.03 (108.42)	0.43
WMHs volume, M ± SD	0.49 (0.53)	0.26 (0.59)	<0.01
Episodic memory function, M ± SD			
LTM Recall	1.67 (0.48)	1.78 (0.45)	0.93
LTM Recognition	2.45 (0.28)	2.45 (0.25)	0.48

Loneliness, Total score for the revised University of California at Los Angeles Loneliness scale; WMHs volume, Log-transformed total volume of white matter hyperintensities; LTM, Long-term memory.

**Table 2:** Pearson’s correlation coefficient between demographic, loneliness, WMHs, memory function, and depressive symptoms.

	Variable	1	2	3	4	5	6	7
1	Gender ^a^							
2	Age	0.106						
3	Education	0.389 ***	−0.163 *					
4	Loneliness	0.095	0.020	−0.03				
5	WMHs volume	0.191 *	0.531 ***	−0.155 *	0.154 *			
6	LTM Recall	−0.094	−0.371 ***	0.349 ***	−0.148 *	−0.404 ***		
7	LTM Recognition	0.021	−0.375 ***	0.503 ***	−0.17 *	−0.421 ***	0.747 ***	
8	Depressive symptoms	0.001	0.118	−0.261 ***	0.297 ***	0.216 **	−0.162 *	−0.261 ***

Correlation represent Pearson’s correlation except for gender; Spearman correlation coefficient represented for gender variable. ^a^ Men: 1, Women: 0. *** *p* < 0.001; ** *p* < 0.01; * *p* < 0.05.

**Table 3:** Mediating effects of loneliness on memory function through WMHs volume.

Model 1	B	SE	t	*p*
Loneliness to WMHs	0.009	0.004	2.20	0.029
WMHs to LTM Recall	−0.183	0.064	−2.844	0.005
Loneliness to LTM Recall	−0.005	0.003	−1.446	0.150
	Effect	SE	Lower, 95% CI	Upper, 95% CI
Indirect effect	−0.002	0.001	−0.0041	−0.0001
F	10.305 ***			
R^2^	0.298			
MSE	0.157			
**Model 2**	**B**	**SE**	**t**	***p***
Loneliness to WMHs	0.009	0.004	2.20	0.029
WMHs to LTM Recognition	−0.115	0.033	−3.458	<0.001
Loneliness to LTM Recognition	−0.003	0.002	−1.748	0.082
	Effect	SE	Lower, 95% CI	Upper, 95% CI
Indirect effect	−0.001	0.001	−0.0022	−0.0001
F	16.274 ***			
R^2^	0.408			
MSE	0.042			

All reported *p* values were two-tailed. Bootstrapping with 95% confidence intervals; B = Unstandardized coefficients; SE = Standard error. *** *p* < 0.001.

**Table 4:** Results of the moderation model.

DV	IV	B	SE	t	*p*
WMHs volume	(Constant)	−2.918	0.561	−5.206	<0.001
	Loneliness	0.004	0.006	0.788	0.431
	Gender	−0.031	0.312	−0.098	0.922
	Loneliness × Gender	0.007	0.008	0.871	0.385
	Age	0.044	0.006	7.385	<0.001
	Education	−0.013	0.009	−1.291	0.198
	Marital status	−0.011	0.111	−0.096	−0.230
	Social network size	0.016	0.027	0.574	0.567
	Interaction frequency	0.0004	0.0004	1.003	0.317
**DV**	**IV**	**B**	**SE**	**t**	***p***
LTM recognition	(Constant)	2.848	0.241	11.815	<0.001
	WMHs volume	−0.089	0.040	−2.241	0.026
	Gender	−0.004	0.051	−0.068	0.946
	WMHs volume × Gender	−0.071	0.060	−1.189	0.236
	Age	−0.007	0.003	−2.414	0.017
	Education	0.020	0.004	6.653	<0.001
	Marital status	−0.033	0.048	−0.677	0.499
	Social network size	0.002	0.012	−0.135	0.893
	Interaction frequency	<0.001	0.0002	0.240	0.810
**DV**	**IV**	**B**	**SE**	**t**	***p***
LTM recall	(Constant)	2.852	0.457	6.240	<0.001
	WMHs volume	−0.073	0.076	−0.965	0.336
	Gender	0.015	0.096	0.151	0.880
	WMHs volume × Gender	−0.270	0.114	−2.373	0.018
	Age	−0.015	0.006	−2.681	0.008
	Education	0.035	0.008	4.234	<0.001
	Marital status	−0.072	0.092	−0.775	0.439
	Social network size	−0.016	0.022	−0.706	0.482
	Interaction frequency	−0.0003	0.0003	−0.956	0.341

**Table 5:** Moderated mediation effects of gender on the relationship between loneliness, WMHs volume, and LTM recall index.

Dependent Variable: LTM Recall Index	B	SE	t	*p*
(Constant)	3.041	0.488	6.23	<0.01
Loneliness	−0.004	0.003	−1.1	0.27
WMHs volume	−0.067	0.076	−0.88	0.38
Gender	0.016	0.096	0.16	0.87
WMHs volume × Gender	−0.259	0.114	−2.27	0.03
Age	−0.016	0.006	−2.77	<0.01
Education	0.034	0.008	4.16	<0.01
Marital status	−0.071	0.092	−0.78	0.44
Social network size	−0.019	0.023	−0.82	0.41
Interaction frequency	−0.0004	0.0004	−1.09	0.28
F	8.677 ***			
R^2^	0.324			
MSE	0.154			

*** *p* < 0.001.

**Table 6:** A conditional indirect effect of gender when WMHs volume mediated the association between loneliness and LTM Recall index.

Gender	Estimates	SE	95% CI	
			Lower	Upper
Female	−0.001	0.001	−0.002	0.001
Male	−0.003	0.002	−0.007	−0.0001

Bootstrap sample size = 5000.

## Data Availability

The data presented in this study are available upon request from the corresponding author. The data are not publicly available for privacy reasons; the data contain sensitive information about the participants.
